# Effective connectivity analysis of response inhibition functional network

**DOI:** 10.3389/fnins.2025.1525038

**Published:** 2025-04-07

**Authors:** Monica Di Giuliano, Andy Schumann, Feliberto de la Cruz, Pedro Henrique Rodrigues Da Silva, Karl-Jürgen Bär

**Affiliations:** ^1^Lab for Autonomic Neuroscience, Imaging and Cognition (LANIC), Department of Psychosomatic Medicine and Psychotherapy, Jena University Hospital, Jena, Germany; ^2^Institute of Psychiatry of the Hospital das Clínicas of the Faculty of Medicine of the University of São Paulo, Ribeirão Preto, Brazil

**Keywords:** response inhibition, dynamic causal modeling, spatial independent component analysis, Go-NoGo task, impulsivity, functional networks

## Abstract

**Introduction:**

Inhibition mechanisms are essential in daily life, helping individuals adapt to environmental demands. However, the causal interactions between large-scale functional networks involved in response inhibition remain poorly understood.

**Methods:**

In this study, we examined the effective connectivity (EC) underlying inhibitory processes in the brain using dynamic causal modeling (DCM) and independent component analysis (ICA). We conducted a Go-NoGo fMRI task with 19 healthy participants to investigate these networks.

**Results:**

Our results identified four functional networks activated during correct motor response inhibition: the salience network (SN), the right and left executive control networks (ECNs), and the ventral default mode network (vDMN). We observed a significant causal inhibitory influence from the vDMN to the left ECN (lECN). Under conditions of unsuccessful response inhibition, the SN, bilateral ECNs, and somatomotor network (SMN) were found to be prominently activated. Furthermore, we identified a significant correlation between the inhibitory influence from the SMN to the SN and the commission error rate. Finally, correlation analyses between self-reported impulsivity levels and causal network interactions revealed that highly impulsive individuals require greater interhemispheric integration between the right and left ECNs for effective inhibition, as well as a causal excitatory modulation from the right executive control network (rECN) to the vDMN.

**Discussion:**

In summary, our study reveals complex hierarchical dynamics among functional networks during response inhibition. These findings offer valuable insight into the neural mechanisms supporting inhibition and provide avenues for future research on the neural underpinnings of this critical cognitive function across the lifespan.

## 1 Introduction

Response inhibition is a multifaceted process, characterized by three main dimensions: a cancellation mechanism, which involves stopping an ongoing response and is typically measured using the Stop-Signal Task (SST); a withholding process, which involves stopping a prepared but not yet initiated response, which is commonly measured using the Go-NoGo task; and finally, an interference resolution function, which is the process of selecting information relevant to an ongoing task while suppressing the processing of irrelevant information, which is typically tested using Stroop, Simon, Flanker, and Antisaccade tasks (Dambacher et al., 2014; Zhang et al., 2017). Overall, these cognitive processes play a critical role as executive control mechanisms supporting future-oriented goals (Miller and Cohen, 2001).

The prefrontal cortex is widely recognized as a crucial cortical hub for response inhibition. However, the localization of activated regions within the frontal cortex varies across studies, with this variation appearing to be task-dependent (Fuster, 1991; Wager et al., 2005; Mostofsky et al., 2003; Simmonds et al., 2008). This region exerts top-down control to determine which stimulus–response associations should be activated in a given context (Zhang et al., 2017; Curtis and D'Esposito, 2003). Substantial evidence supports a dorsal/ventral dissociation within the prefrontal cortex during response inhibition, indicating that ventral lateral prefrontal regions (VLPFC) are involved in the maintenance of information, while the dorsolateral prefrontal cortex (DLPFC) is responsible for manipulating information in working memory (WM) (Simmonds et al., 2008; Curtis and D'Esposito, 2003). Given its critical role in the domain of WM, the prefrontal region is essential for guiding response inhibition, particularly under conditions of increased working memory demand (Zhang et al., 2017; Simmonds et al., 2008; Curtis and D'Esposito, 2003).

Focusing on the widely implemented Go-NoGo task, the prefrontal region is not the only pivotal hub sustaining response inhibition. The cortical–subcortical nodes involved in response inhibition encompass the supplementary motor area (SMA), left premotor cortex, bilateral inferior parietal region, bilateral occipital regions, putamen, and bilateral insula (Buchsbaum et al., 2005). Overall, all of these regions are implicated in the processes of stimulus recognition, maintenance, and manipulation during the selection of the motor response (Curtis and D'Esposito, 2003; Rubia et al., 2001; Corbetta and Shulman, 2002; Bellgrove et al., 2004; Blasi et al., 2006).

In modern neuroscience, it is well established that the brain is organized into large-scale functional networks. Cortical–subcortical regions form specific functional networks that regulate low-frequency fluctuations underlying resting states and influence cognitive task performance. Widely recognized functional networks include the default mode network (DMN), the salience network (SN), and the executive control network (ECN). The DMN mediates emotional processes, self-referential mental activity, and spontaneous cognition (Park and Friston, 2013; Raichle, 2015). This network can be divided into a dorsal subsystem (e.g., dorsal medial prefrontal cortex, precuneus, and posterior cingulate cortex) and a ventral subsystem (ventral medial prefrontal cortex, posterior inferior parietal lobule, retrosplenial cortex, and medial temporal pole). The ECN, on the other hand, comprises the dorsolateral prefrontal cortex and posterior parietal cortex hubs, is primarily activated when a cognitively demanding task requires attention and top-down control processes (Goulden et al., 2014; Fox et al., 2006). Finally, the SN involves the ventral lateral prefrontal cortex, anterior insula, and anterior cingulate cortex seed regions (Raichle, 2015; Seeley et al., 2007). The SN responds when an emotional, cognitive, or homeostatic stimulus captures attention, regulating both the internal and external attentional processes (Raichle, 2015; Seeley et al., 2007). Particularly during motor tasks, a widely studied functional system is the somatomotor network (SMN). This network comprises cortico-striato-thalamo circuits involved in skeletomotor control, with main hubs located in the precentral and postcentral gyri, as well as the SMA (Kozlowska et al., 2018; Wieclawski et al., 2024). The SMN serves multiple functions, including orchestrating motor responses such as error detection, motor initiation, inhibition, and feedback (Kozlowska et al., 2018; Wieclawski et al., 2024).

The top-down and bottom-up hierarchies of large-scale networks can be investigated by assessing the causal (directional) relationships underlying their connectivity patterns, highlighting the importance of effective connectivity (EC) (Park and Friston, 2013). EC describes the causal influence of one neuronal population on another, reflecting specific models of causal dynamics (Park and Friston, 2013). A widely used method for inferring these causal network interplays is dynamic causal modeling (DCM). DCM aims to uncover the causal architecture of coupled or distributed dynamical systems by defining a causal model characterized by interregional connections and self-connections within each brain region (Park and Friston, 2013). Thus, DCM enables the inference of the causal synaptic strength of connections between and within regions of interest (Friston et al., 2003). Combining independent component analysis (ICA) and DCM is a promising approach to investigating causal connectivities among different large-scale functional networks. ICA separates data into spatially independent patterns of activity, enabling the identification of brain networks engaged in a task or resting-state conditions without imposing predefined regions of interest/priors when specifying DCM models (Hidalgo-Lopez et al., 2021).

Specifically, multiple networks appear to be involved in response inhibition; however, their causal interactions are largely unknown (Stevens et al., 2007; Erika-Florence et al., 2014). To the best of our knowledge, only one recent study has employed both DCM and ICA approaches to infer causal connectivities between brain regions identified based on a spatially distributed and aggregated pattern of activity during successful response inhibition (Stevens et al., 2007). Through DCM model specification and the estimation of the ECs between these regions, the study highlighted distinct anatomical areas that exhibit task-related activation patterns during the stopping of planned but not yet initiated motor responses (withholding). Hierarchical patterns of EC are described, where the causal and dependent interplay between fronto-striatal-thalamic, premotor, and frontal–parietal regions is crucial for response suppression (Stevens et al., 2007). Specifically, the fronto-striatal-thalamic regions regulate both direct and indirect modulations of other brain components to achieve successful withholding of a prepotent response (Stevens et al., 2007). These findings align with another study that implemented an ICA approach to extract time series from regions correlated with a stop-signal task. The authors highlighted that spatially distributed brain activations occur during the cancellation-inhibition processes (Erika-Florence et al., 2014). In this context, the insular, opercular, premotor, and anterior cingulate regions are identified as key components for processing infrequent and novel stimuli that should be suppressed (Erika-Florence et al., 2014). Overall, these studies suggest that behavioral inhibition arises from different localized brain components that are not solely confined to frontal modules, forming a holistic neuronal system for multiple cognitive demands during the inhibition mechanisms (Stevens et al., 2007; Erika-Florence et al., 2014). Nevertheless, focusing on spatially distributed regions to parameterize withholding models may be somewhat limiting. This perspective involves aggregating various remote and localized brain regions based on their activation patterns across different conditions or tasks. Thus, emphasis is placed on regions where activity is integrated from various areas grouped into discrete or localized regions. Therefore, more studies should focus on the relationship between large-scale networks, which are considered widespread brain systems characterized by functional connections or correlated functional activity patterns. This approach would provide a solid scientific foundation for understanding how functional network modulations orchestrate correct or incorrect response inhibition as a crucial cognitive function. Thus, to the best of our knowledge, a deep understanding of the EC (directional) interplay between established large-scale functional networks (e.g., DMN, SN, and ECN) during response inhibition is timely. In fact, EC between large-scale functional networks is primarily investigated during resting-state fMRI designs (Li et al., 2020).

To bridge this gap, we used Dynamic Causal Modeling (DCM) to assess the causal influences of functional networks related to response inhibition during a Go-NoGo task in a healthy population. We hypothesize that the main top-down and bottom-up networks play different roles during a successful withholding response compared to an incorrect inhibition of the motor response. Specifically, we aim to test the top-down and bottom-up causal connectivities between large-scale functional networks that affect the correct and incorrect response inhibition mechanisms. Our study thus addresses the neural mechanisms underlying the dynamics of the withholding process. Understanding the dynamics could lead us to propose a new perspective as a starting point, which may serve as a target endophenotype for future clinical interventions (Abramovitch et al., 2015; Slaats-Willemse et al., 2003; Wylie et al., 2007; Collantoni et al., 2016; Glass et al., 2011; Kaladjian et al., 2011; Sneed et al., 2007).

## 2 Materials and methods

### 2.1 Participants

The participants consisted of 19 healthy individuals (9 men and 10 women; mean age = 27.6 years, SD = 6.5) were recruited from the local community and interviewed by a medical research assistant. Individuals with a history of drug use, any medical conditions, neurological or psychiatric disorders, and/or first-degree relatives with Axis I psychiatric disorders were excluded from participation. All subjects were native German speakers, right-handed according to the modified version of Annett’s Handedness Inventory (Briggs and Nebes, 1975), and provided written informed consent before participation. To assess and better understand the general neuropsychological profile of our sample, we utilized a set of self-report questionnaires, including the State–Trait Anxiety Inventory (STAI-S/T) and the Barratt Impulsiveness Scale-11 (BIS-11). Main anamnestic and subclinical scores, means, and standard deviations are reported in Table 1 (Laux et al., 1981; Patton et al., 1995). The Ethics Committee of the University of Jena approved the study protocol. All subjects were compensated with €8 per hour for their participation.

**Table 1:** Main demographics and subclinical data.

General	Mean ± standard deviation
Age (years)	27.6 ± 6.5 (18 to 47)
Sex	M = 9; *F* = 10
Education	No = 0
	Primary = 1
	Secondary = 1
	Higher level = 17
Neuropsychological assessments	
STAI-S	36.8 ± 8.1 (22 to 50)
STAI-T	38.5 ± 8.9 (20 to 50)
BIS-11 total score	58.5 ± 11.3 (44 to 87)
BIS-11 Non-Planning subscale	21.4 ± 3.9 (17 to 30)
BIS-11 Attentional-Impulsivity subscale	16.5 ± 4.4 (10 to 27)
BIS-11 Motor-Impulsivity subscale	20.7 ± 4.5 (14 to 31)

Mean ± standard deviation values, including their range, are reported for demographic and subclinical variables. M, male; F, female; STAI-S/T, State–Trait Inventory; BIS-11, Barratt Impulsiveness Scale-11.

### 2.2 Go-NoGo task design

The Go-NoGo paradigm is a commonly used task for measuring the ability to inhibit a prepotent response (Leimkuhler and Mesulam, 1985). The NoGo signal, which triggers the inhibitory processes, is presented unexpectedly following a Go signal, assessing the inhibition of a planned response (action restraint; Eagle et al., 2008). Our research group developed a modified version of the task, which is described in detail in our previous study (Köhler et al., 2018). Typically, the task is weighted toward the Go stimuli to build a prepotent tendency to respond, thereby increasing the inhibitory effort required to successfully withhold responses to NoGo stimuli. In short, at the beginning of the experiment, participants saw the word “READY” in the middle of the screen. The word “READY” was then replaced by a clay jug from which water frequently dripped, representing our baseline measure.

After varying time intervals, a stimulus appeared, which was either a Go or a NoGo trial. The Go stimulus included two types of transverse cracks originating from either the left side or the right side of the jug. The NoGo stimulus consisted of two types of vertical cracks originating from either the upper end or the bottom end of the jug. The stimuli were presented for 600 ms. The inter-stimulus intervals (ISI) were 3,800, 6,000, and 8,200 ms, presented sequentially and equally, with corresponding stimulus-onset asynchronies (SOAs) of 4,400, 6,600, and 8,800 ms. Initially, the stimulus presentation onsets were delayed by 0 to 1.6 s as additional temporal jitter to prevent the water drops from serving as the stimulus timing and to enhance the temporal resolution of the hemodynamic responses. Subsequently, immediately after the stimulus presentation, water continued to drop into the jug: there were more Go stimuli (∼74% of cases) than NoGo stimuli (∼26% of cases). This imbalance enabled us to create a prepotent response tendency and better distinguish the cognitive withholding mechanism in suppressing the planned but not yet initialized motor response.

All subjects were asked to indicate which type of crack was presented by either pressing a button (with their right index finger) as quickly as possible when a Go stimulus appeared or by restraining their response when a NoGo stimulus appeared. In the present study, we adapted the experiment in terms of total duration and structure. Thus, in this study, the Go-NoGo experiment lasted approximately 23 min, featuring 160 Go and 42 NoGo stimuli. Performance was assessed by the number of correct reactions in both the Go and NoGo conditions. In summary, the benefits of implementing a revised version of the task follow this rationale: the Go-NoGo task typically relies on frequent and fast-paced Go trials to establish pre-potency. However, the BOLD signal measured during fMRI is relatively slow, with the peak hemodynamic response occurring approximately 5 s after the stimulus onset. To better resolve BOLD responses to the presented trials, we increased the inter-stimulus interval while maintaining pre-potency. The scenario of water drops falling into a jar until it breaks was chosen as a relatable and intuitive representation for participants. Between trials, the falling water drops were intended to establish motor readiness. Additionally, the Go trials were presented more frequently. In earlier studies, this task was used while recording BOLD activations and physiological responses, which corroborated its basic functionality (Köhler et al., 2018).

### 2.3 Image acquisition and processing

Data were collected using a 3 T whole-body system equipped with a 64-element receive-only head matrix coil. T2*-weighted images were obtained through a gradient-echo echo-planar imaging (EPI) sequence (TR = 2,120 ms, TE = 36 ms, TA = 2,100 ms, FOV = 224 mm^2^, acquisition matrix = 160 × 160 mm2, flip angle = 90°) with 104 interleaved transverse slices of 1.4 mm thickness, a multi-band acceleration factor of 4, and an in-plane resolution of 1.4 × 1.4 mm^2^. A series of 606 whole-brain volume sets were acquired in one session lasting approximately 23 min. High-resolution anatomical T1-weighted volume scans (MPRAGE) were captured in sagittal orientation (TR = 2,300 ms, TE = 3.03 ms, TI = 900 ms, flip angle = 9°, FOV = 256 mm × 256 mm, matrix = 256 × 256, number of sagittal slices = 192, acceleration factor (PAT) = 2) with an isotropic resolution of (1 × 1 × 1) mm^3^. Data analysis was conducted using SPM12.^1^ The first four images were discarded to ensure a steady-state tissue magnetization condition. Further preprocessing steps of the fMRI data included slice timing correction, rigid body realignment to the mean of all images, and alignment of functional and anatomical data. Subsequently, images were normalized to MNI space and smoothed with a Gaussian kernel of 4 mm full-width at half-maximum.

### 2.4 Spatial ICA analysis

We opted for a multivariate, data-driven approach to explore response inhibition as a brain function. In this context, we conducted a spatial ICA. The ICA methods are available in the Group ICA of fMRI Toolbox (GIFT v1.3b) implemented in MATLAB.^2^ One of the first steps was to define the dimensionality reduction method, and we chose a Minimum Description Length Criterion (MDL) algorithm for model selection, approximation, and signal complexity reduction. MDL was used to avoid potential order selection biases that could hinder ICA in estimating the dimension of the signal subspace in fMRI data (Calhoun and de Lacy, 2017). Specifically, MDL allows for the selection of an optimal model to approximate the data with minimal complexity (Calhoun and de Lacy, 2017). For the number of independent components (ICs) to extract, we adopted a standard figure of 20. A range of 20–50 components is typically reported in most studies; depending on the data, higher model orders yield more focal components, whereas lower model orders result in larger networks (Calhoun and de Lacy, 2017). Finally, by using a low-order model, we differentiated between various lateralized functional networks and discarded noise components through visual inspection of each IC (Calhoun and de Lacy, 2017). Furthermore, to address the randomness inherent in the initialization of the ICA algorithm decomposition, we combined two specific ICA algorithms: ICASSO and Infomax. ICASSO performs the analysis multiple times with different random initializations and subsequently quantifies the consistency of the outcomes. The ICASSO software package is used for the quantitative evaluation of the stability of ICA estimation at different selected orders. Conversely, Infomax is an ICA algorithm designed to maximize information criteria by enhancing mutual information (Calhoun and de Lacy, 2017). Data were reduced through two principal component analysis (PCA) stages (first subject-specific PCA = 25; second group-based = 20) and concatenated at each stage for further reduction. The concatenated data from the reduction step were used to aggregate ICA components and back-reconstruct component time courses and spatial maps for each participant (group-ICA/GICA back reconstruction step). The aggregated components and the results from data reduction are used to compute individual subject components. The resulting single-subject time course amplitudes were then calibrated (scaled) using the raw data to reflect the percentage of fMRI signal change for comparison across participants. The individual back-reconstructed components were then used to compute a mean spatial map and time course, a standard deviation spatial map and time course, and a t-statistic spatial map (Stevens et al., 2007).

### 2.5 Functional network identification

The time course analysis involved parameterizing the time courses of each IC using a temporal multiple regression. We then estimated the association between each component’s time course and two conditions that characterize the Go-NoGo experimental design. The hemodynamic response results from three different conditions in the task design: correct rejects, false alarms, and hits. In line with our research aims, we used the design matrices for correct response inhibition (correct rejects) and for incorrect response inhibition (false alarm errors) as two temporal regressors, which we previously estimated with a first-level General Linear Model (GLM). This convolution model’s multiple regression analysis yielded *R*^2^ values that represented the overall association of each condition in the experimental design with each component’s time course. We examined the mean *β*-weights, showcasing the coupling of each component to the experimental conditions (correct rejects and false alarm errors) using one-sample *t*-tests against zero. Component *p*-values that differed significantly from zero indicated an association with that condition. We visually inspected these t-statistical maps produced by the GICA analysis for each significant IC. The detection and identification of each functional network were compared with ROIs in the MNI space commonly found in the literature (Abramovitch et al., 2015; Slaats-Willemse et al., 2003; Wylie et al., 2007; Collantoni et al., 2016; Glass et al., 2011; Kaladjian et al., 2011; Sneed et al., 2007). Additionally, we compared literature studies on large-scale network definitions with our visual inspection of each functional network by leveraging a built-in automatic labeling function of the GIFT-ICA toolbox, which serves as a spatial correlation of each IC with predefined functional network templates (Abramovitch et al., 2015; Slaats-Willemse et al., 2003; Wylie et al., 2007; Collantoni et al., 2016; Glass et al., 2011; Kaladjian et al., 2011; Sneed et al., 2007).

### 2.6 Dynamic causal modeling

The aim of dynamic causal modeling (DCM) is to infer the causal architecture of coupled or distributed dynamical systems. DCM posits a causal model in which neuronal activity in a given region influences changes in neuronal activity in other regions through interregional connections, as well as in its own activity through self-connections (Friston et al., 2003). Additionally, any of these connections can be modulated by contextual variables. Importantly, the parameters are constrained to align with *a priori* specifications regarding the range within which they are likely to fall. These constraints, formulated as a prior distribution, are then combined with data through a likelihood distribution to establish a posterior distribution according to Bayes’ rule. Changes in EC can then be inferred using Bayesian inference based on the posterior densities (Calhoun and de Lacy, 2017; Li et al., 2007; Calhoun et al., 2001). In this study, DCM was implemented to estimate the EC couplings and decouplings between each functional network detected for each Go-NoGo condition. To achieve this goal, we first estimated the global maximum activation in each IC’s time map to extract the eigenvariate representative of each network, which will be used to compute the DCM model specification. We defined these coordinates as the centers of the ROI masks. This procedure enabled us to extract, for each condition and subject, the volumes of interest (VOIs) necessary for the specification and estimation of the DCM models. Subject-specific coordinates were identified within an 11 mm radius of the group-level peak within these region-specific masks. This procedure follows a pipeline already implemented in the context of the ICA-DCM approach to extract IC time series, which can be used as input for DCM models (Hidalgo-Lopez et al., 2021). The time series from these ROIs were then extracted for each subject, creating the VOIs. Our primary focus was on EC between ROIs in the left and right hemispheres. The inter-hemispheric differences fall outside the scope of this study. DCM operates on a hypothesis-driven model space specification, which requires some parameter specifications. For Bayes model specification, we implemented a DCM time-series model, as this technique is commonly used in fMRI task designs. We opted for a bilinear model space with a two-state specification, allowing connections to remain unconstrained—neither exclusively excitatory nor exclusively inhibitory—without stochastic effects (i.e., no random influences). We explored all possible inter-regional and intra-regional modulations between each network by comparing a fully connected model with a null/control model (where only matrix A is fully connected and activated). In this model space, we needed to specify prior parameters in matrix A, which are context-independent and represent fixed EC among brain regions mediated by anatomical connections; matrix B, which represents context-dependent changes in EC induced by the task (u1); and matrix C, which represents context-dependent driving inputs of the task (u2) that modulate the activation of each node (Gelman et al., 1995). The order of each entry (VOIs) for each matrix is specific and driven by the hypothesis of modeling top-down and bottom-up forward/backward connections between each network, considering the roles these functional networks play during the cognitive control processes (Simmonds et al., 2008).

Then, we conducted Bayesian Model Estimation to identify parameters (e.g., connection strengths) that provide the best trade-off between explaining the data and minimizing complexity (i.e., keeping parameters close to their prior or initial values). Model estimation combines priors with observed fMRI data to produce updated posterior beliefs. According to Bayes’ rule, the posterior distribution equals the likelihood multiplied by the prior, divided by the evidence (Gelman et al., 1995). Next, Bayesian Model Selection (BMS) was performed to identify the best-fitting model that explains the EC framework for each condition, using a Random Effects Analysis (RFX). In this analysis, the error variance is estimated from the variability of subject-specific effects across participants, enabling a population-level generalization of our results while balancing data fit and model complexity. The RFX results are summarized by the following parameters: the exceedance probability (xp), which assumes that each model has a different frequency within the group; the Bayesian Omnibus Risk (BOR), which indicates the probability that all models share the same frequency within the group; the protected exceedance probability (pxp), which adjusts xp using the BOR and was recently introduced as a summary statistic (Rigoux et al., 2014). These findings quantify the probability that any one model is more frequent than the others beyond chance.

Given that numerous parameters can best explain our model of evidence, one method to reduce and select the optimized parameters based on BMS and comparison is Bayesian Model Reduction. In brief, Bayesian Model Reduction enables the evaluation of model evidence when the model is simplified by removing one or more parameters. A key aspect of Bayesian model reduction is that this evidence can be evaluated using the posteriors and priors of a parent model that includes all potential parameters. Clearly, there are a vast number of parameter combinations that could be considered. Since Bayesian model reduction evaluates the effect of altering the precision of priors on model evidence, it can be described as an automatic Bayesian sensitivity analysis (Wasserman, 2000; Friston et al., 2018).

#### 2.6.1 Bayesian model averaging and analysis of network dynamics

Based on the winning models derived from the two separate RFX analyses for each condition, we aimed to parameterize the specific intra-regional modulation and inter-regional modulation strengths within and between each node in the three matrices by implementing Bayesian Model Averaging (BMA). Specifically, BMA eliminates the dependence of parameter inference on the selected model, considering the entire model space (or an optimal winning model/family of models) and computing the weighted averages of each model parameter, with the weights determined by the posterior probability for each model (Berger, 2011; Hoeting et al., 1999). We used BMA to compute the average parameter estimates across the winning models from the previous Bayesian Model Selection and Comparison step and then statistically compared these averages using one-sample *t*-tests on the input pathway coefficients to extract EC parameters that were significantly different from 0, after adjusting for the Bonferroni-Holm family-wise error method (0.05). The main results targeted for the *t*-test and the multiple comparison correction were the posterior average expectations (mEP) and the posterior expectations standard deviation (sEP). These parameters were calculated in both group-wise and subject-wise manners.

### 2.7 Correlation between DCM parameters and behavioral data

To determine whether the DCM parameters accurately predicted performance in both correct and incorrect response inhibition conditions, we regressed the modulation parameters of the winning model against the behavioral data across participants. We examined two types of behavioral data: the number of correct rejects and the number of false alarms. Accordingly, we conducted a Spearman correlation analysis (*p* = 0.05) between each coefficient BMA parameterized in the winning model matrices and the number of correct and incorrect response inhibitions. Simultaneously, we correlated the BMA pathway coefficients with the total scores obtained from the Barratt Impulsiveness Scale-11 (BIS-11). The *p*-values were uncorrected for multiple comparisons, as these analyses form part of an exploratory approach.

## 3 Results

**Figure 1:**
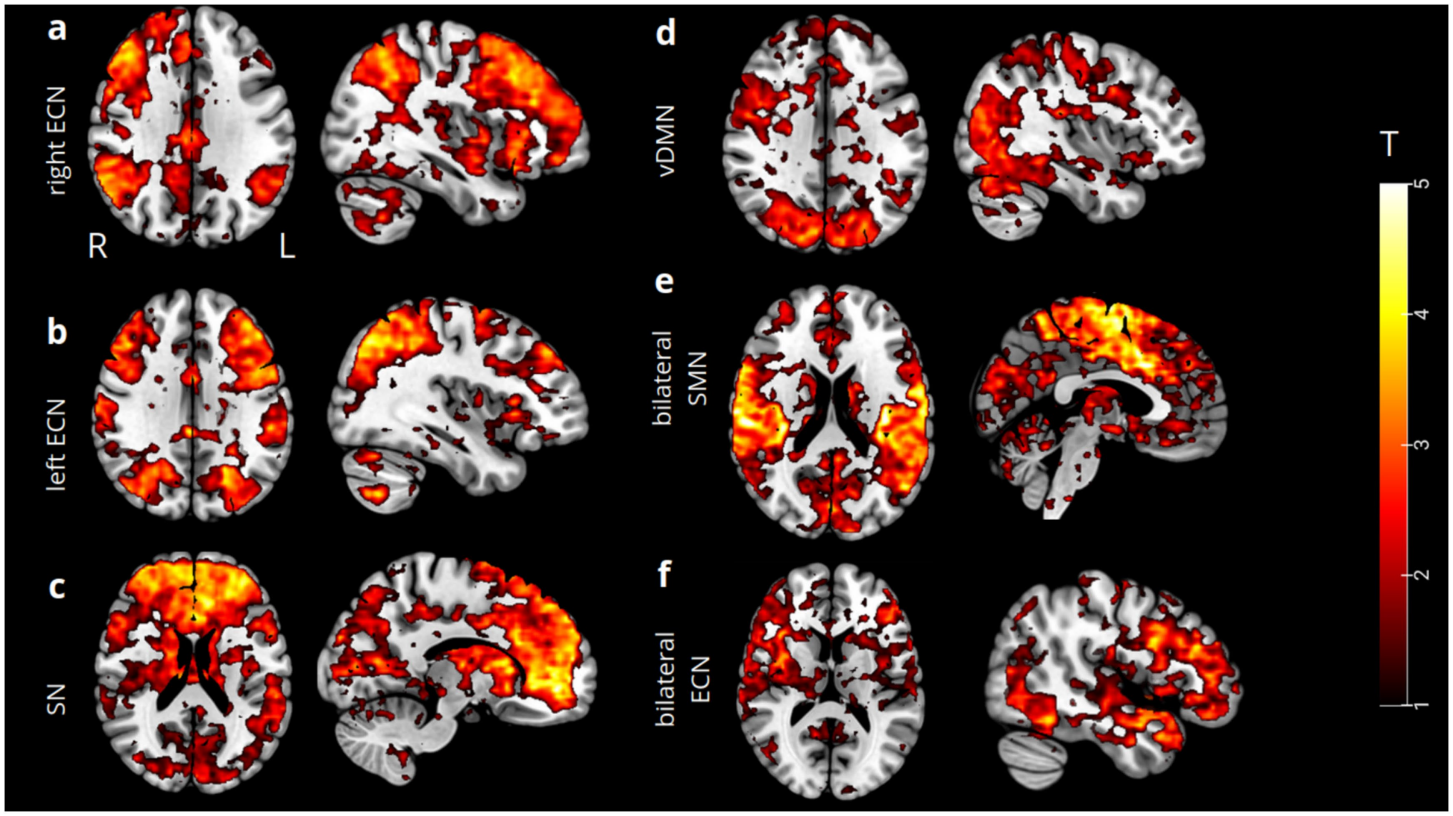
Spatial ICA t-maps (thresholded at *p* = 0.05) reassembling our functional networks resulting from correct and incorrect response inhibition showed that certain brain regions exhibited statistically significant activation during both task conditions. These regions were labeled as follows: the right ECN **(a)**, left ECN **(b)**, bilateral SN **(c)**, and left vDMN **(d)**. Conversely, regions that exhibited statistically significant activation during incorrect response inhibition were labeled as bilateral SMN **(e)** and bilateral ECN **(f)**, including bilateral SN.

The spatial ICA yielded 20 ICs. Four of these components were identified as being primarily associated with successful response inhibition performance (*p* = 0.05): the right executive control network (rECN), the left ECN, the bilateral SN, and the left ventral default mode network (vDMN). Conversely, the functional networks mainly associated with incorrect response inhibition included (*p* = 0.05) the bilateral SN, the bilateral ECN, and the bilateral SMN. Each component defines a distinct functional network (Figure 1), which we visually inspected and compared to established functional templates available in the literature (Abramovitch et al., 2015; Slaats-Willemse et al., 2003; Wylie et al., 2007; Collantoni et al., 2016; Glass et al., 2011; Kaladjian et al., 2011; Sneed et al., 2007). Simultaneously, we utilized an automated labeling function in the GIFT-ICA toolbox, which facilitates the spatial correlation of each IC with predefined functional network templates (Seeley et al., 2007; Li et al., 2020; Habas et al., 2009; Raichle, 2011; Razi et al., 2017). We then implemented a dynamic causal modeling (DCM) approach. We selected four regions identified by the global maximum activation in each functional network’s t-statistical map (see Methods), corresponding to correct response inhibition: the right inferior temporal gyrus (rITG) for the right ECN (x = 60; y = −25; z = −22); the left inferior frontal gyrus, pars opercularis (lIFG) for the left ECN (x = −44; y = 18; z = 26); the right anterior prefrontal cortex (aPFC) for the SN (x = 12; y = 48; z = −6); and the left medial temporal gyrus (lMTG) for the vDMN (x = −50; y = −32; z = −8). For incorrect response inhibition, the following regions were noted: the left inferior frontal gyrus, pars triangularis (lIFG) for the ECN (x = −38; y = 36; z = 2); the left supplementary motor area (lSMA) for the SMN (x = 0; y = −6; z = 64); and ultimately, the same coordinates for the SN as in the correct response inhibition condition.

We first established the endogenous (matrix A), task-modulatory (matrix B), and driven-effect (matrix C) inputs within our Bayesian model space by selecting subject-specific volumes of interest (VOIs). The VOIs represent the global maximum activation across each functional network. The Bayesian model specification and estimation procedures produced two bilinear models for each condition: one fully connected for all matrices and another serving as a control, in which only the priors in matrix A are fully connected.

Then, we conducted Bayesian model selection and comparison (BMS) using a random effects analysis (RFX) between the two specified models to identify the posterior distribution that best fits our data. The analysis favored the fully connected model over the null model. Specifically, for correct response inhibition, the winning model exhibited an expected probability (exp) of 0.95, an exceedance probability (xp) of 1, and a protected exceedance probability (pxp) of 1. Similarly, for incorrect response inhibition, the winning model showed an exp. of 0.95, an xp of 1, and a pxp of 1.

We then investigated the intra-regional and inter-regional modulations, as well as the effect of each task condition on the excitatory/inhibitory state of the nodes present in the model space. By applying a Bayes Averaging Model (BMA), we eliminated redundant parameters, providing a clearer explanation for the differences among our connectivity parameters. At the same time, the BMA parameterized the EC of the single coefficient (posterior densities). During correct response inhibition, we observed a significant decoupling between the ventral DMN and the left ECN (pFWE = 0.04), indicating an inhibitory forward decoupling from the ventral part of the DMN to the left ECN when a successful withholding of response is executed. Simultaneously, the task condition during which subjects correctly inhibited their motor response positively activated all the nodes representing each functional network, resulting in an excitatory effect on them (for all networks, pFWE <= 0.001).

**Figure 2:**
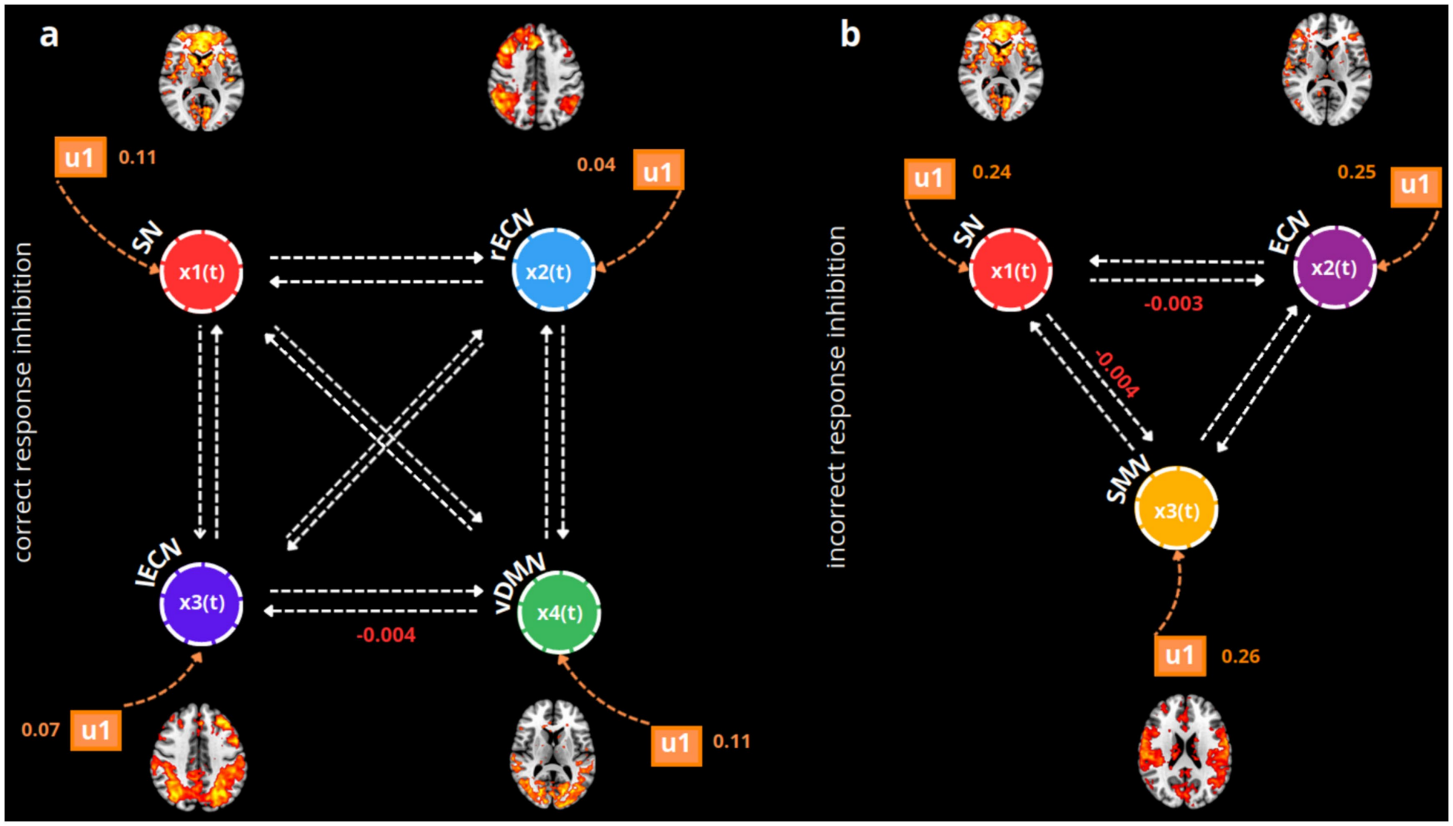
BMA posterior densities of the winning model (fully connected) during correct **(a)** and incorrect response inhibitions **(b)**. For simplicity, only the statistically significant coefficients (mean of posterior probabilities) estimated using a one-sample *t*-test are displayed for matrix B (modulatory inputs, indicated by white arrows, u2) and matrix C (the driving effect of the task, indicated by orange arrows, u1). Each functional network is represented as an xn(t) neuronal node in the model, using different colors: red for the SN, blue for the rECN, violet for the left executive control network (lECN), green for the vDMN, light violet for the bilateral executive control network (ECN), and yellow for the SMN. For both winning fully connected models, the u1 and u2 modulatory inputs are shown with means of posterior distributions that are significant according to the one-sample *t*-tests (with Bonferroni-Holm correction, *p* = 0.05).

Instead, when the subjects incorrectly inhibit their motor responses during the NoGo trials, a decoupling can be observed between the SN and the bilateral ECN (pFWE = 0.01), as well as between the SN and the SMN networks (pFWE = 0.02). Inhibitory forward modulation of the SN is then outlined on both the ECN and the SMN. Notably, the specific incorrect NoGo condition drives positive and excitatory activation in the SN (pFWE = 0.03), in the bilateral ECN (pFWE = 0.009), and in the SMN (pFWE <= 0.001). In Figure 2, the most significant BMA coefficient posterior densities (coupling/decoupling) for both conditions are shown.

**Figure 3:**
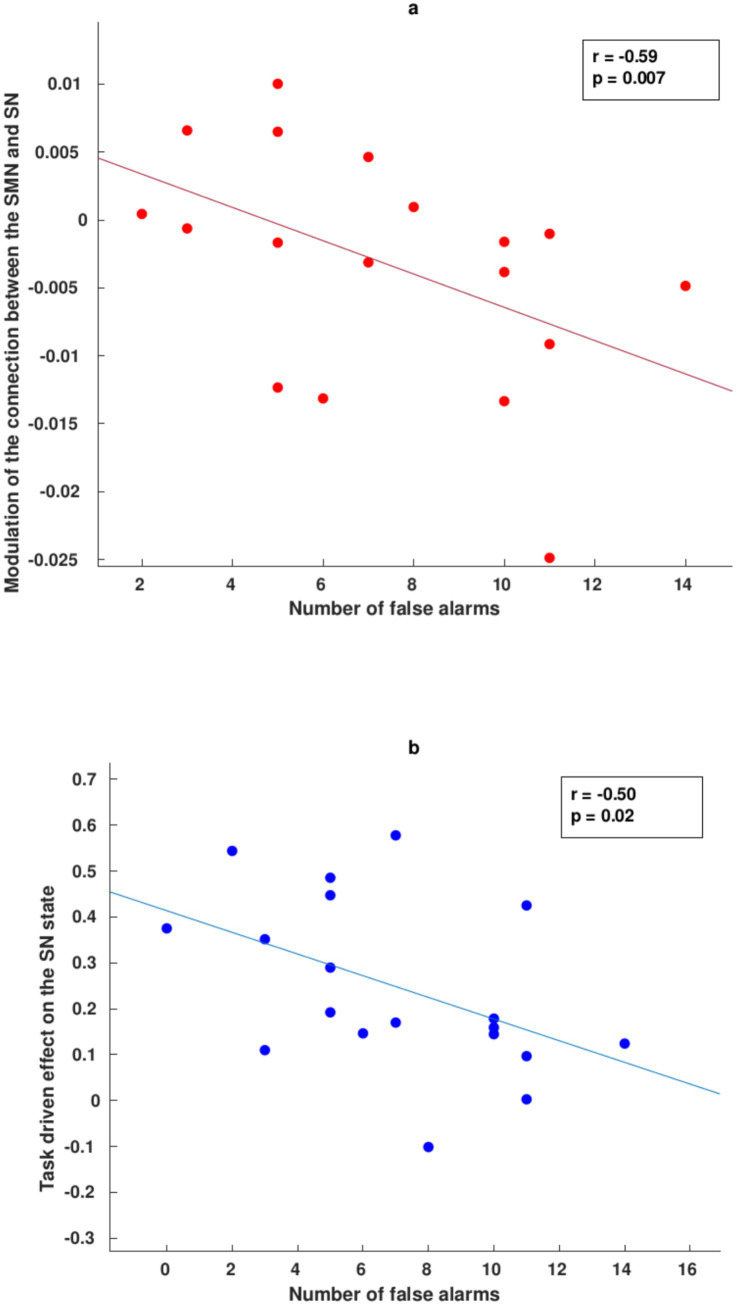
Correlation between DCM parameters and behavioral data during incorrect response conditions. **(a)** Negative correlation between the modulation of EC from the SMN to the SN (decoupling) and the number of incorrect NoGo responses. **(b)** Negative correlation between the task’s driving effect on the excitatory state of the SN and the number of false alarms.

We finally investigated the behavioral relevance of the winning fully connected models specified for each condition. During the accurate response conditions, we did not find a significant correlation between the vDMN and lECN decoupling and the number of correctly rejected trials (*R*^2^ = 0.31, *p* = 0.1 uncorrected). Incidentally, no driven activations of the task condition on each node were found to be significantly correlated with the number of accurately withheld responses (SN, *R*^2^ = −0.16, *p* = 0.4 uncorrected; rECN, *R*^2^ = 0.11, *p* = 0.6 uncorrected; lECN, *R*^2^ = 0.009, *p* = 0.9 uncorrected; vDMN, *R*^2^ = −0.27, *p* = 0.2 uncorrected). Regarding the number of incorrect response inhibitions (Figure 3), we found a significant negative correlation between the SMN and SN decoupling modulation (*R*^2^ = −0.59; *p* = 0.007, uncorrected), as well as a negative correlation regarding the task-driven effect solely on the excitatory state of the SN (*R*^2^ = −0.50, *p* = 0.02 uncorrected). No significant correlation was found regarding the relevant forward inhibition from the SN to the ECN (*R*^2^ = 0.19, *p* = 0.4 uncorrected) or to the SMN (*R*^2^ = 0.19, *p* = 0.4 uncorrected).

**Figure 4:**
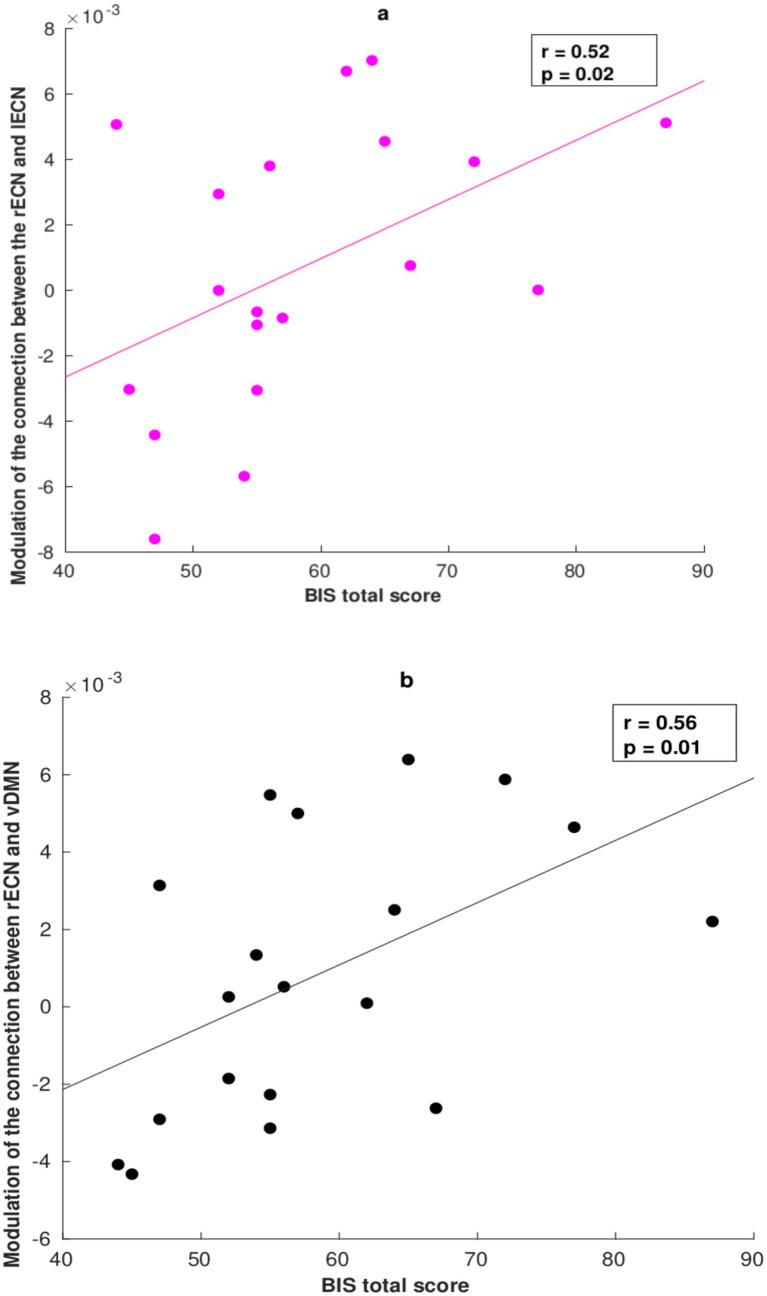
Correlation between the DCM parameter and Barratt Impulsiveness Scale (BIS-11) total score during the correct response condition. **(a)** Positive correlation between the modulation of EC from the rECN to the lECN (coupling) and the total score obtained from the BIS-11. **(b)** Positive correlation between the modulation of EC from the rECN to the vDMN (coupling) and the total score on the BIS-11.

Ultimately, we applied a Spearman correlation analysis to examine the association between the modulatory connectivities and the effects driven by the task conditions in relation to the Barratt Impulsiveness Scale (BIS-11). The correlation results between the EC parameters of the two conditions and the other three subscales of the BIS-11 questionnaire (Non-Planning, Attention-Impulsivity, and Motor-Impulsivity) are better detailed in the Supplementary materials. Specifically, we found a significant correlation between the effective parameters and the total BIS score only during the correct NoGo task condition (Figure 4): a positive association was found between the forward coupling from the rECN to the lECN and the total score obtained from the BIS-11 questionnaire (*R*^2^ = 0.52, *p* = 0.02, uncorrected) and the same was observed for the forward coupling between the rECN and vDMN (*R*^2^ = 0.56, *p* = 0.01, uncorrected).

## 4 Discussion

This study was conducted to characterize the EC of large-scale functional networks that play significant roles in both correct and incorrect response inhibitions. To the best of our knowledge, prior evidence regarding the networks contributing to successful response inhibition has primarily been inferred from studies focused on spatially distributed and localized brain regions aggregated based on activation patterns across different task conditions (Stevens et al., 2007; Erika-Florence et al., 2014).

From this starting point, our study aims to explore which well-established large-scale functional networks—defined as widespread brain areas characterized by correlated functional activation patterns (e.g., DMN, ECN, and SN)—play a crucial role in both successful and unsuccessful response inhibition. Furthermore, we aimed to model and quantify the causal interactions between these different functional networks across each Go-NoGo condition.

### 4.1 Causal interactions of functional networks in correct response inhibition

During correct response inhibition conditions, we identified several major functional networks that play important roles: the SN, the executive control networks (ECNs), and the vDMN. The SN regulates bottom-up attention to external and internal stimuli (Uddin, 2015). Thus, the emotional and perceptual salience of these stimuli could lead to functional coupling between the SN and the ECNs, especially when attention to salient information requires top-down regulation (Uddin, 2015; Zabelina and Andrews-Hanna, 2016).

These two networks were found to be positively activated during the correct withholding condition, suggesting an excitatory effect on the involved nodes for this task. The IC maps of these two networks revealed widespread activation in the prefrontal, insular-opercular, dorsal anterior cingulate, striatal basal ganglia, parietal, and temporal regions for the SN, with peak global activation observed in the aPFC. Additionally, the bilateral ECNs exhibited extensive activation in the dorsolateral prefrontal and parietal regions, along with lateralized activations in the temporal, cingulate, and thalamic areas.

A recent finding highlights the excitatory driving effect of the task on the ventral counterpart of the DMN. Unlike the SN and the ECN, the DMN is a well-known network associated with internally directed cognition (Raichle, 2015). The DMN can be divided into two main subsystems. The first subsystem, termed the dorsal subsystem, includes the dorsal-medial prefrontal cortex and overlaps with other core regions of the DMN, such as the precuneus and the posterior cingulate cortex. The second subsystem, termed the “ventral DMN,” consists of the ventral medial prefrontal cortex, posterior inferior parietal lobule, retrosplenial cortex, parahippocampal cortex, hippocampal formation, and medial temporal pole (Raichle, 2015). The dDMN is involved in introspective, self-oriented processes, while the ventral DMN engages in decision-making and cognitive control processes (Raichle, 2015; Greicius et al., 2003; Shirer et al., 2012). Based on the findings we outlined regarding correct response inhibition, we hypothesized a differentiation between cognitive control processes for preparing to inhibit a response versus fully inhibiting a response. Indeed, during correct response withholding, a significant modulation of decoupling is observed between the vDMN and the left ECN (lIFG, pars opercularis). These findings suggest an inhibitory function originating from the vDMN toward the left ECN, revealing a specific temporal dynamic that may be necessary during NoGo trials (Simmonds et al., 2008; Raichle, 2015; Greicius et al., 2003; Shirer et al., 2012; Andrews-Hanna et al., 2010). In this context, one of the central hubs of the vDMN is located in the MTG area (Raichle, 2015; Greicius et al., 2003; Shirer et al., 2012). Bilateral middle temporal functional activations are more strongly associated with fully inhibiting a response compared to completing a response in the Go-NoGo complex variant (Simmonds et al., 2008; Goghari and MacDonald III, 2009; Badre et al., 2005). Specifically, according to other studies, the MTG is sensitive to stimulus–response mappings (Anderson et al., 2016). Additionally, the MTG plays a crucial role depending on the semantic strength of the stimulus that needs to be retrieved and updated (Simmonds et al., 2008; Anderson et al., 2016). Therefore, an initial mapping of the stimulus may be necessary to retrieve and update the memory system when a complex stimulus is presented, varying across trials. This function, guided by the vDMN, could facilitate the adaptive and successful selection of the motor response. Consequently, we propose that the vDMN could serve as a circuit “breaker” for top-down networks during NoGo processing (like the ECN), enabling adaptive goal-contingency disengagement in the early stages of response inhibition before the selection of a competing response (Anderson et al., 2016). This implies that the causal modulation of the vDMN would enable targeting and assessing the stimulus in light of current predefined goals; however, once selected, its correlated response should subsequently be ignored when response-irrelevant stimuli are presented (Anderson et al., 2016; Esterman et al., 2013; Weissman et al., 2006; Krmpotich et al., 2013). The finding related to significant inhibition and decoupling between the vDMN and the left hemispheric counterpart of the ECN could be deemed relevant in the context of the hemispheric specialization processes during cognitive control tasks (Habas et al., 2009; Shirer et al., 2012; Damoiseaux et al., 2006; Brass and Haggard, 2008). The left ECN is more involved in approach behaviors, described as reaching goals through a willingness to pursue a reward. In contrast, studies suggest that the right ECN counterpart is more involved in avoidance behaviors (Krmpotich et al., 2013). Following our results, avoidance behavior is essential for accurately preventing the subject from engaging in a motor-reward approach directed toward responding to the stimulus.

Another possible interpretation is that individuals with high interhemispheric specialization are better at withholding their responses during NoGo conditions. This theoretical framework could enhance our understanding of not only the inhibition from the vDMN to the lECN but also the correlation findings related to the BIS-11 total score. In this context, we observed that only during correct NoGo conditions, lower impulsivity in responding to the NoGo stimulus correlates with decreased coupling between the two contralateral areas in the ECN. Conversely, based on our findings, as individuals become more impulsive, greater interhemispheric integration between the rECN (rITG) and lECN is required. Normally, a surplus of interhemispheric connections promotes functional balance by ensuring an even distribution of cognitive processes across the two hemispheres (Martínez et al., 2018; Serrien et al., 2006). This mechanism is particularly true when more cognitive resources are necessary to perform a specific task (Martínez et al., 2018; Serrien et al., 2006). Nevertheless, during a particular cognitive task, the hemisphere with a higher number of interconnected brain hubs involved in that task may downregulate activation in the less active hemisphere to optimize task performance (Martínez et al., 2018; Serrien et al., 2006). Despite the necessity for hemispheric dominance during specific tasks, interhemispheric specialization is evolutionarily adaptive to support overall cognitive processes (Martínez et al., 2018; Serrien et al., 2006). The right hemisphere (RH) is organized more efficiently, exhibiting greater regional interconnectivity than the left hemisphere (LH), which tends to incorporate more central hubs (Brass and Haggard, 2008; Martínez et al., 2018; Serrien et al., 2006; Corbetta et al., 1993; Mesulam, 1999; Wenderoth et al., 2004). In terms of cognitive functions supported by the RH, its specialization may relate to controlling spatial attention for both the left and right visual fields, or it may serve a monitoring function that becomes particularly important in conflict situations, such as when there is a mismatch between motor intention, proprioception, and/or visual feedback (Martínez et al., 2018; Serrien et al., 2006; Corbetta et al., 1993; Mesulam, 1999; Wenderoth et al., 2004; Fink et al., 1999). Thus, adaptive motor behavior may depend on inhibitory processes associated with right hemispheric specialization, along with the facilitatory processes that promote the integration of information across both hemispheres; this integration may depend on individual cognitive resources and impulsivity traits (Serrien et al., 2006). Indeed, impulsivity is a multidimensional construct that describes a tendency to act without forethought in response to internal or external stimuli (Wenderoth et al., 2004; Fink et al., 1999). Impulsivity in younger individuals appears to be associated with a more widespread, less efficient organizational connectivity of brain networks (Fornaro et al., 2024). Thus, it is plausible that right lateralization during correct response inhibition of major ECN activation could favor successful inhibition of motor responses in individuals with lower impulsivity traits who require less interhemispheric integration. Conversely, during correct NoGo conditions, those exhibiting a higher impulsivity profile necessitated stronger integration between the right and left areas of the ECN to successfully inhibit their motor response (Moeller et al., 2001; Dalley and Robbins, 2017). At the same time, the positive correlation between successful motor response inhibition and the coupling between the rECN and vDMN might be interpreted within this theoretical framework. Individuals with less impulsivity traits may be able to inhibit competitive responses to NoGo stimuli, with the vDMN serving as a modulatory and preliminary step in stimulus mapping and monitoring. In contrast, individuals characterized by a stronger impulsivity profile favored an excitatory causal modulation from the rECN to the vDMN as an additional cognitive control process, which might be needed throughout the entire inhibition mechanism (Slaats-Willemse et al., 2003; Wylie et al., 2007; Collantoni et al., 2016; Glass et al., 2011; Dalley and Robbins, 2017; Garavan et al., 1999; Garavan et al., 2002; Bogacz et al., 2010; Mulder et al., 2014; Wolpe et al., 2022; Chao et al., 2009; Baranger et al., 2023; Campos et al., 2005; Wang et al., 2019; Jenkins et al., 2000; Kwon and Kwon, 2013; Swann et al., 2012; Rae et al., 2015). We believe that future studies are needed to clearly distinguish the role of each part of the ECN during cognitive control tasks and to determine if hemispheric specialization—similar to that observed in the ECN—exists during these types of mechanisms.

### 4.2 Causal interactions of functional networks in incorrect response inhibition

Regarding the incorrect response inhibition condition, we observed significant activation in all functional networks involved in this process (SN and bilateral ECN), including the SMN network, which we discovered only in this specific condition. Specifically, the SMN IC map covers distributed premotor and motor areas, along with activations in the parietal, temporal, precuneus, thalamic, and cerebellar regions, with the global maximum activation located in the lSMA. In contrast, dorsolateral prefrontal and parietal activations were primarily found in the bilateral IC map of the ECN, along with key regions for temporal, cingulate, striatal, and insular-opercular activations. Furthermore, we observed a significant decoupling between the SN network (aPFC) and the bilateral ECN (lIFG) as well as the SMN network, suggesting that inhibition from the SN is implicated in modulating the other networks. This finding highlights the SN’s role in attentional control over the ECN and SMN during the transition from processing internal to external salient information. Recognizing specific salient features of a stimulus—such as NoGo stimuli—may lead to attempts to inhibit a motor response (Garavan et al., 1999; Garavan et al., 2002). Nevertheless, another consistent finding is the positive driven effect exercised by the task condition not only on the ECN and SN state, but also on the SMN. This network activation may explain why inhibition from the SN is not always effective in restraining a motor response and in influencing decision-making processes involved in selecting appropriate behaviors. The SMN has been shown to regulate voluntary action by modulating decision thresholds—whether, when, and which action to perform (Bogacz et al., 2010). The SMA hub within this network determines the appropriate attentional threshold for choosing an action: in the context of stopping a motor response, elevating individual attentional thresholds could support a more cautious strategy for delaying the motor response. Conversely, lower attentional thresholds could result in rapid and automatic responses, potentially leading to erroneous actions (Mulder et al., 2014; Wolpe et al., 2022; Chao et al., 2009). In cases of incorrect response inhibition, complex NoGo trials may have contributed to misleading individual attentional and cognitive thresholds, resulting in inappropriate response withholding. These findings could be further enhanced by the role of the SMN in the reward anticipation processing mechanisms. Central brain hubs of the SMN in the motor cortex (e.g., pre-SMA/SMA)—along with other nodes in functional networks (e.g., dorsal and ventral attention, SN)—are correlated with reward-driven behaviors (Baranger et al., 2023). Notably, while the primary function of the SMN is sensory and motor processing, studies indicate that motor areas may also be involved during the reward anticipation and expectancy processes, as SMN areas contribute to initiating motor responses (Baranger et al., 2023; Campos et al., 2005; Wang et al., 2019). Importantly, stimulus–reward associations can influence sensory processing, which, in turn, can strongly modulate attention functions to optimize goal-directed behavior (Wang et al., 2019). Thus, accelerated reward anticipation—guided by the central hubs of the SMN—could have led to quicker motor responses favoring immediate rewards, potentially at the expense of response inhibition (Baranger et al., 2023; Wang et al., 2019). Future studies in healthy individuals should further explore the role of SMN, along with other functional networks, in supporting response inhibition mechanisms due to its involvement in the reward and attentional processes.

Moreover, these results align with the findings from the correlation analysis examining the number of false alarms associated with the interaction between each causal connectivity. Specifically, the less effectively the SN facilitates adaptive switching between internally and externally oriented attention, the more impulsively subjects commit errors (false alarms) during this condition. Specifically, our findings suggest that the SN is not capable of effectively regulating and controlling the activation of the SMN during incorrect response inhibition conditions. In this context, we found that an increase in errors corresponds to a decreased ability of the SN to actively coordinate cognitive and attentional control over the subjects’ motor responses. Indeed, we discovered a significant correlation between the number of false alarms and the causal inhibition exerted by the SMN on the SN. Thus, we propose that the more the SMN exerts causal inhibition on the SN, the less capable the individuals are of successfully performing motor response inhibition (Chao et al., 2009; Baranger et al., 2023; Campos et al., 2005; Wang et al., 2019; Jenkins et al., 2000; Kwon and Kwon, 2013). Notably, when withholding an ongoing action, the SMN is crucial for transmitting information to functional networks—such as the SN and ECN—that are necessary for successfully controlling subjects’ impulses to respond to a non-salient stimulus (Swann et al., 2012; Rae et al., 2015). This can be viewed as a preparatory step for delaying motor responses, which is primarily needed to generate active inhibition of goal-directed behaviors (Jha et al., 2015; Allen et al., 2018; Mostofsky and Simmonds, 2008). In the context of our study, we speculate that the causal connectivity from the SMN to the SN is significant in how individuals respond to NoGo stimuli: the causal modulation driven by the SMN may reduce the SN’s cognitive and attentional control functions over motor responses (Mostofsky and Simmonds, 2008).

### 4.3 Response inhibition during the course of the lifespan: future implications

In conclusion, we would like to highlight the relevance of our current findings and their link to the dimensional development of inhibition functions from healthy to pathological states. Specifically, age-related declines in response inhibition are frequently reported in the literature, with a higher number of suppression errors due to a general decline in attentional and mnemonic functions throughout the lifespan (Gibson et al., 2018). During adolescence, the connectivity between frontal–parietal and frontal-striatal-thalamic regions correlates with the number of false alarms or incorrect response inhibition mechanisms. These findings suggest a reduced engagement of the brain areas responsible for the top-down regulation of inhibition processes, which become more pronounced and specialized in adulthood (Stevens et al., 2007). Overall, age-related differences are observed in functional connectivity between the frontal–parietal and frontal-striatal-thalamic areas, possibly reflecting increased “top-down” executive control to compensate for weaker anatomical connections or incomplete functional specialization (Stevens et al., 2007). Nevertheless, throughout the lifespan, the majority of studies indicate a sharp decline in the suppression of motor responses, generally correlating with the integrity of frontal areas during aging (Gibson et al., 2018; Mather and Carstensen, 2005; Borella et al., 2008). Specifically, the decline in inhibition processes over the lifespan has been proposed as selective rather than indicative of general cognitive decline, depending on the type of inhibition dimension being investigated (Borella et al., 2008; Rey-Mermet and Gade, 2018). In Go-NoGo tasks and suppression mechanisms, a recent meta-analysis revealed that, as age increases, older adults are less capable of maintaining and coordinating the NoGo/stop-signal information compared to young adults due to age-related deficits in working memory and attention. These cognitive deficits indicate selective inhibition impairments that occur over the lifespan rather than an overall decline in suppressing dominant responses (Rey-Mermet and Gade, 2018). Decreased engagement of frontal and posterior parietal regions in response to increasing working memory load is often observed in this context, coupled with failures to suppress task-irrelevant activity in regions encompassing midline DMN regions and the premotor/motor area (Rieck et al., 2021). Given the role of the hierarchical top-down and bottom-up functional network causal connectivities revealed in our current study, which support the role of the ventral DMN and SMN in regulating withholding functions, future research should replicate and investigate these connectivity patterns from healthy to pathological states. This research would provide cutting-edge insights into potential brain biomarkers explaining goal-directed/reward-driven behaviors and inhibition mechanisms throughout the lifespan (Rieck et al., 2021).

## 5 Limitations

Several caveats must be considered in our study. First, the small sample size presents a potential limitation for the statistical power of the analyses. Second, the model specification in DCM, which significantly influences the interpretation of further results, is crucial for accurately identifying the model that best fits the data. Future studies should focus on different specifications of Bayes-family models, utilizing more nodes/eigenvariates to represent each functional network. In this context, we chose one eigenvariate to serve as the representative for each functional network. This choice is motivated by a similar methodology previously implemented in relevant DCM studies in the field, aimed at extracting the time series of ICs that can be used as input for DCM model specification and estimation (Hidalgo-Lopez et al., 2021). Nonetheless, given the exploratory nature of this study, we used only one node to represent large-scale functional networks resembling the IC map. Considering this caveat, future studies should explore more refined methods to represent entire functional networks, fitting more complex DCM models with a higher number of priors to better illustrate the causal interactions of functional networks. Third, future studies should place greater emphasis on hemispheric specialization as a potential factor in explaining cognitive control processes, such as response inhibition. In this context, a more thorough understanding of the psychological profile of a healthy population could provide better insights into the complex interactions between impulsivity traits and cognitive control mechanisms. This purpose could serve as a fundamental starting point for future studies involving clinical samples as well as aging populations characterized by high levels of impulsivity traits.

## 6 Conclusion

In conclusion, the combination of ICA and DCM analysis allowed us to consider the canonical top-down cognitive streams and the reciprocal influence of bottom-up pathways originating from the vDMN and subcortically from the SMN nodes. These non-canonical functional pathways are essential for accounting for the primary preparation steps that occur during memory retrieval and in updating the competitive signal stimuli, as well as mediating the withholding of motor responses. Furthermore, the lateralization of correct decision-making processes during the inhibition of motor responses—associated with low levels of impulsivity traits—may be significant in terms of hemispheric specialization. Studying cognitive control processes, such as response inhibition in healthy populations, represents merely the first step in establishing the theoretical and methodological foundations needed to understand how impulsivity and incorrect cognitive control mechanisms could serve as vital endophenotypes across various disorders (Abramovitch et al., 2015; Slaats-Willemse et al., 2003; Wylie et al., 2007; Collantoni et al., 2016; Glass et al., 2011; Kaladjian et al., 2011; Sneed et al., 2007). We ultimately suggest that future studies explore more complex EC patterns characterizing large-scale functional networks in relation to brain biomarkers, which could explain the complex neuropsychological processes underlying response inhibition.

## Data Availability

The data supporting the fundings of this study are available from the corresponding author upon reasonable request.

## Footnotes

^1^ https://www.fil.ion.ucl.ac.uk/spm

^2^ http://icatb.sourceforge.net
