# Supplementary material for: Effective connectivity analysis of response inhibition functional network
Source: Front Neurosci. 2025 Apr 7;19:1525038. doi: 10.3389/fnins.2025.1525038 (PMC12009941; doi:10.3389/fnins.2025.1525038)
Supplement: Supplementary file 1 [file Data_Sheet_1.docx]

**Supplementary Materials**


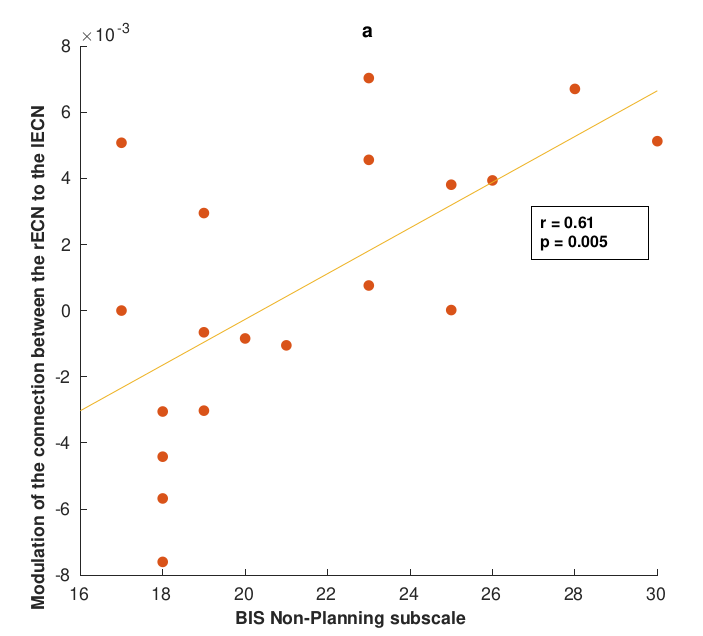


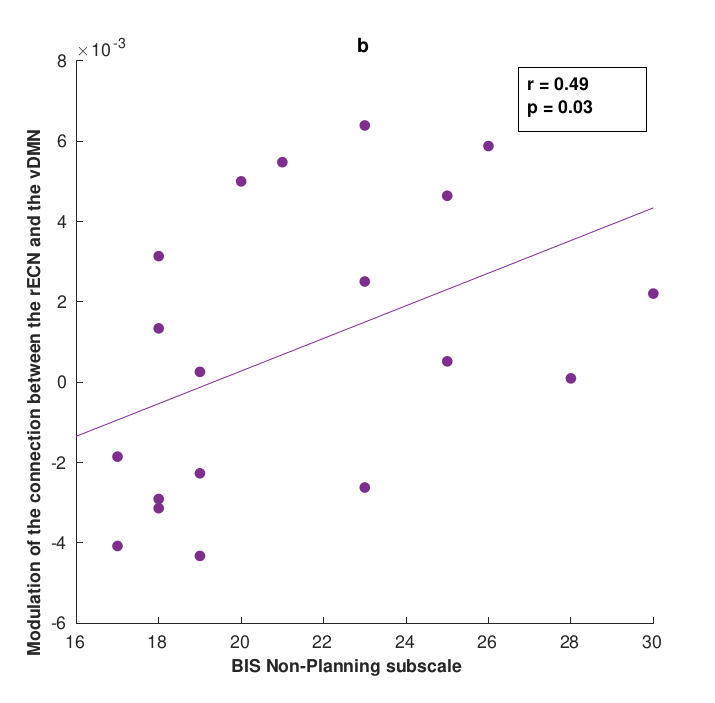


**Fig.S1** Correlation between DCM parameter and Barratt Impulsiveness Scale (BIS-11) Non-Planning (BIS-NP) subscale during correct response condition. **a.** Positive correlation between the modulation of the effective connectivity from the rECN to the lECN (coupling) and the BIS-NP total score. **b.** Positive correlation between the modulation of the effective connectivity from the rECN to the vDMN (coupling) and the BIS-NP total score**.**


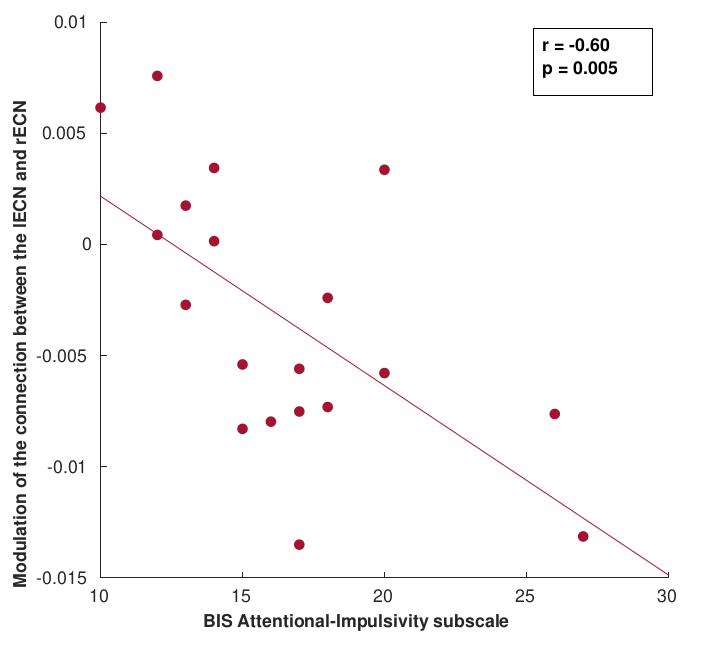


**Fig.S2** Correlation between DCM parameter and Barratt Impulsiveness Scale (BIS-11) Attentional-Impulsivity (BIS-A) subscale during correct response condition. Negative correlation between the modulation of the effective connectivity from the lECN to the rECN (decoupling) and the BIS-A total score.


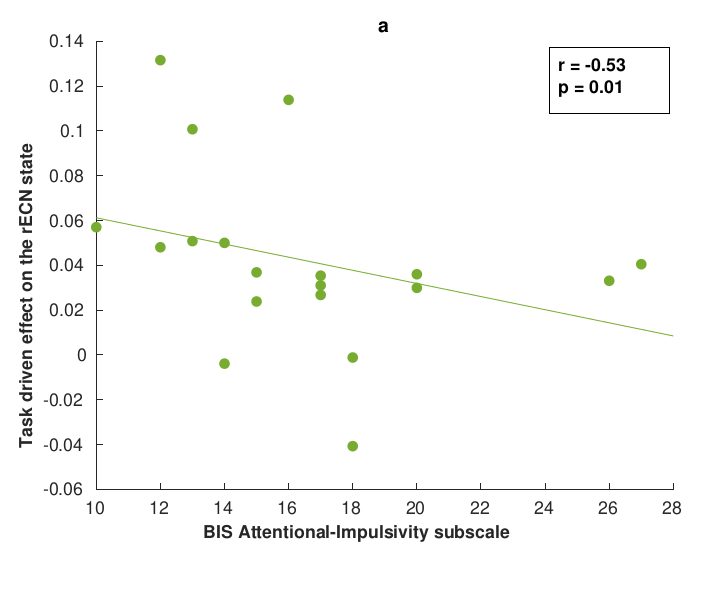


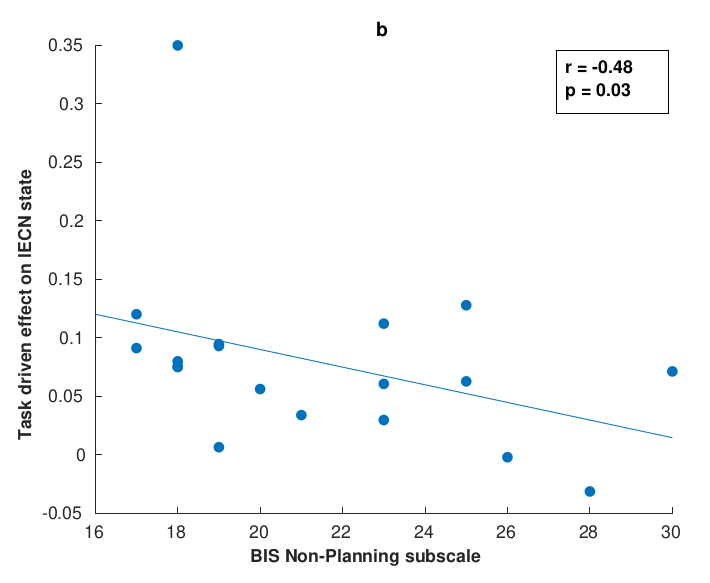


**Fig.S3** Correlation between DCM driving effect parameter and Barratt Impulsiveness Scale (BIS-11) Non-Planning (BIS-NP) subscale and Attentional-Impulsivity (BIS-A) subscale during correct response condition. **a.** Negative correlation between BIS-A total score and task driving effect on the excitatory state of the rECN. **b.** Negative correlation between the total score at BIS-NP and task driving effect on the excitatory state of the lECN**.**


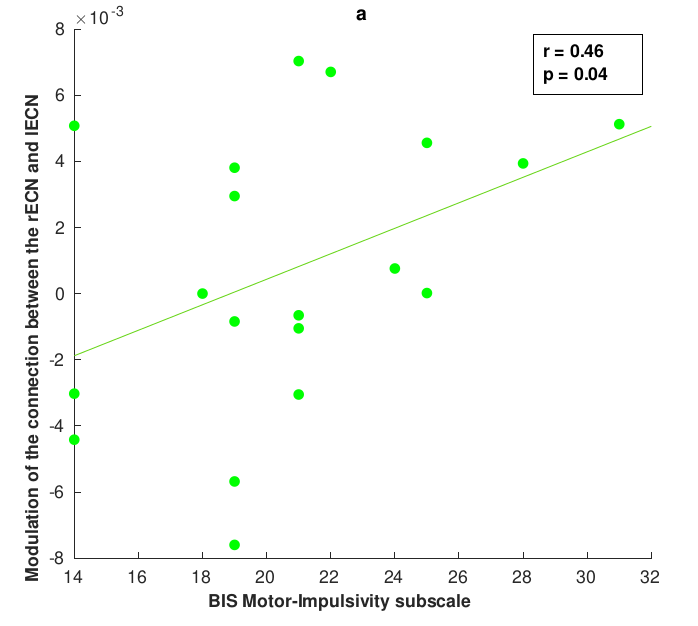


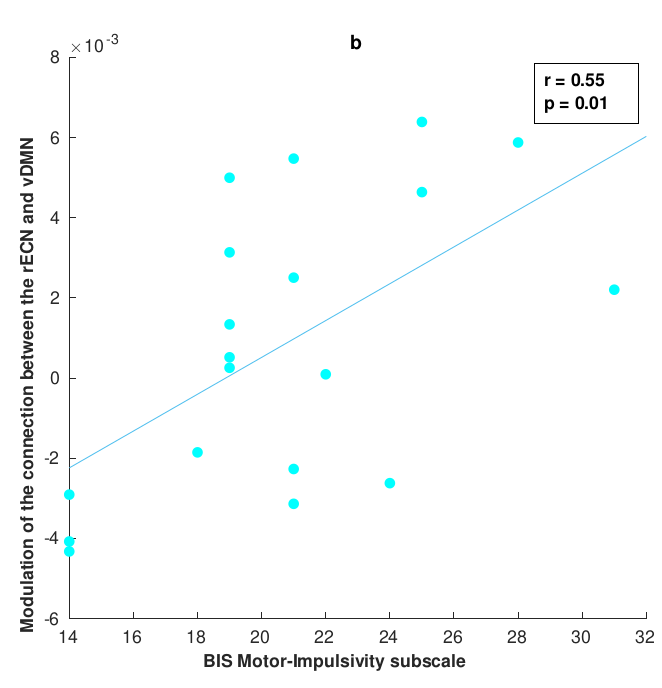


**Fig.S4** Correlation between DCM driving effect parameter and Barratt Impulsiveness Scale (BIS-11) Motor-Impulsivity (BIS-M) subscale during correct response condition. **a.** Positive correlation between the modulation of the effective connectivity between rECN and lECN (coupling) and the BIS-M total score. **b** Positive correlation between the modulation of the effective connectivity between rECN and vDMN (coupling) and the BIS-M total score.
